# Working Harder at Working Together: Building Collaboration between Public Health and Health Care Delivery

**DOI:** 10.3389/fpubh.2015.00167

**Published:** 2015-07-10

**Authors:** Chris Van Gorder

**Affiliations:** ^1^Scripps Health Corporate Office, San Diego, CA, USA

**Keywords:** collaboration, lessons learned, leadership, communication

In recent years, health care providers across the spectrum have begun to knit back together a system that has become excessively and dangerously fragmented. My own organization, Scripps Health^1^, has been working to connect health care data with other public and private providers through regional health information systems^2^. In addition, we are working collaboratively with the San Diego County Health and Human Services Agency on a federally funded project to help patients move more easily between public and private health care providers (1).

Notwithstanding efforts, such as these, it is often difficult for public health practitioners and health care providers to collaborate. Such collaborative efforts should reduce inefficiencies in the greater healthcare system, result in improved responses to public health crises, and improve the support of public health. By developing improved organizational structures in both public health and health care organizations, as well as by improving communication between the two, collaboration can and will improve, but in doing so, it will require strong leadership at all levels on both sides of the public health/health care delivery divide. As we consider the Scripps Health experience in collaborating with public health organizations, we should remember that the definition of public health is broad, encompassing the prevention of epidemics and disease, protection against environmental hazards, prevention of injuries, promotion of healthy behaviors and mental health, disaster and recovery assistance for communities, and provision of accessible and quality health services (2).

Scripps Health has had a range of experiences engaging with public health practitioners, most notable in response to disasters. During Hurricane Katrina, Scripps answered the call from the Surgeon General of the United States and sent a medical response team to care for the evacuees, treating 500 patients a day at one point in Houston. Other teams of physicians and nurses assisted area clinics in treating survivors. In Houston, I supported and protected our teams, putting to use my training as a former police officer, and describing our efforts to proud employees at home (3).

We responded again in 2010, when a massive earthquake devastated the country of Haiti. We called for Scripps volunteers and almost 2,000 employees answered, resulting in two response teams being sent to provide medical care to desperate survivors. Before our teams left Southern California, I assessed the conditions in Haiti to determine opportunities for us to be of assistance, and to assure the safety of our volunteers. What I saw stunned me: people suffering from horrific injuries, and medical personnel lacking such basics as oxygen, anesthetic drugs, antibiotics, and blood. I had the opportunity to recall my EMT training when asked to assist with a surgical procedure; leaders need to show front-line staff that they are both capable of and willing to undertake the activities that they are asking of others. Our teams stayed in Haiti for a week, working with clinicians from the University of Maryland to ease the suffering of the earthquake survivors and to help them begin the long healing process.

For us, Katrina and Haiti were not merely incidental opportunities to help improve public health; they were organizational turning points. As employees at home learned how their colleagues responded to these emergencies, their pride in Scripps Health swelled, and the usual “silo” mentality fell by the wayside. More than ever before, we knew that we were all in this together and that we really were One Scripps. I will never forget how our supply chain managers responded to our Katrina response team, “We’re here for you,” and how one of our computer programmers called her co-workers on the team “heroes,” writing, “Be safe and take care. T-E-A-M Together Everyone Accomplishes More” (4).

Opportunities to serve others in times of crisis can serve as key moments to bridge professional differences and bring people together, but the impetus for this must come from leaders, who set the tone. Leaders need to engage, get their hands dirty, and work with others outside their disciplines. They need to articulate a sense of higher purpose that can elevate individuals beyond their narrow interests and familiar circle of colleagues. They also need to serve as documenters of the collaboration, thus allowing their organizations to know the good that occurs when people venture beyond their comfort zones to work together.

If leaders work only for further collaboration during moments of crisis, they are not doing enough. The gospel of collaboration must not only be preached every day on a number of levels but also must be demonstrated and supported through the structures that leaders put in place within their organizations. Individually, leaders can nurture a collaborative mindset simply by venturing not only outside of their offices to engage with employees across their own organizations but also across organizational boundaries (5). Staying in touch with employees is important for many reasons, since it conveys the message that leaders need to move beyond the confines of their four walls or their cubicles to see what their colleagues are doing, and to understand the challenges they face on the job.

At Scripps, as leaders, we have created structures within our organization that break down disciplinary boundaries, developing and deploying multi-disciplinary teams to redesign areas of our operations. In redesigning our emergency rooms, a group of practitioners, including physicians, nurses, technicians, and administrators, worked together for weeks, sharing their points of view, addressing challenges, and arriving at solutions. As part of our structural changes, we have also created a “matrix” organization that brings local and system-wide executives together to make decisions. When people in different disciplines work together, applying a variety of points of view to pressing problems, our entire organization wins.

Communication is a central issue that leaders should address when seeking to enhance collaboration, which will result in bridging existing gaps. When I took over as CEO at Scripps, our organization was beset by conflict between administrators and physicians. Drawing on my earlier training as a police officer, I realized that both sides needed to understand better the other’s point of view, so that the two sides could come together as partners. A formal structure was required in order to make this happen, and the Physician Leadership Council (PLC) was created, charged with advising administrators – up to and including the CEO – on key decisions faced by the organization. Although physicians were skeptical at first, it was not long before collaboration between the PLC and the administration was helping the organization come to compromise solutions that could be accepted by all. The PLC remains an important voice in the running of Scripps, and the organization has yet to reject a PLC recommendation, and the mutual trust that has emerged has enhanced collaboration.

Public health practitioners and health care providers may come from different backgrounds, but I believe that we have far more which unites us than divides us. What is now required is leadership drawn from both. Every opportunity should be seized to build organizational structures that will result in improved collaboration. Leaders of both public health and health care organizations are required to provide positive examples for creating structures that will lead to change.

## Conflict of Interest Statement

The author declares that the research was conducted in the absence of any commercial or financial relationships that could be construed as a potential conflict of interest.

## Footnotes

^1^www.scripps.org

^2^http://sdhealthconnect.org/About_SDRHIE/participants.html
